# Pediatric multiple sclerosis

**DOI:** 10.4103/0972-2327.58281

**Published:** 2009

**Authors:** Yashma Patel, Vikram Bhise, Lauren Krupp

**Affiliations:** Department of Neurology. Stony Brook University Medical Center, Stony Brook, NY 11794 USA

**Keywords:** Pediatric multiple sclerosis

## Abstract

Pediatric multiple sclerosis (MS) represents a particular MS subgroup with unique diagnostic challenges and many unanswered questions. Due to the narrow window of environmental exposures and clinical disease expression, children with MS may represent a particularly important group to study to gain a better understanding of MS pathogenesis. Acute disseminated encephalomyelitis (ADEM) is more common in children than in adults, often making the differential diagnosis of MS, particularly a clinically isolated syndrome, quite difficult. Although both disorders represent acute inflammatory disorders of the central nervous system and have overlapping symptoms, ADEM is typically (not always) self-limiting. The presence of encephalopathy is much more characteristic of ADEM and may help in distinguishing between the two. Young children (under ten years old) with MS differ the most from adults. They have a lower frequency of oligoclonal bands in their cerebrospinal fluid and are less likely to have discrete lesions on MRI. Problems of cognitive dysfunction and psychosocial adjustment have particularly serious implications in both children and teenagers with MS. Increased awareness of these difficulties and interventions are needed. While clinical research on therapies to alter the disease course is limited, the available data fortunately suggests that disease-modifying therapy is well tolerated and likely to be effective. Ultimately, multinational research studies are necessary to advance our knowledge of the causes, symptoms, and treatment of pediatric MS and such collaborations are currently underway.

## Introduction

Pediatric multiple sclerosis (MS) has long been an under-recognized and undertreated MS subgroup. However, awareness has grown of the special diagnostic challenges, the clinical course, and the special needs of these patients in the past ten years. As pediatric MS occurs in most of the world, an International Pediatric MS Study group was created that is committed to clarifying the frequency and treatment patterns of children with MS worldwide. While some aspects of the clinical disease in children resemble those of adults, children can also dramatically differ in clinical, radiological, and laboratory features. The family unit plays a different role in pediatric MS than in adult MS. Management is complicated by the lack of clinical trials in this age group. Nonetheless, experience in evaluating and treating children has rapidly grown and the current review summarizes this experience.

## Demographics

The proportion of patients with MS below the age of 16 years is estimated to be 2.7–5%.[1–4] The frequency is much lower and is estimated to be 0.2–0.7% among young children (age 10 and younger),.[5–9] A prospective study found an incidence of 0.9% of initial demyelinating events (IDE) in childhood.[10] As in adults with MS, pediatric MS displays an overall female preponderance. However, the gender ratio varies with age of onset. In children with an onset before six years, the female to male ratio is almost equal at 0.8:1. But the female to male ratio increases to 1.6:1 for an onset between six and ten years of age and even further to 2:1 for an onset over ten years of age.[11] Some additional aspects of the demographic profile of pediatric MS differ from those of adults with MS. A higher proportion of African-Americans was found in the pediatric onset category compared to the adult onset MS group (7.4 *vs*. 4.3%) in an outpatient center in Boston.[8] Others have noted greater ethnic diversity and ancestry in pediatric MS compared to adult MS.[12,13] A positive family history of MS is seen in 6–20% of the children with the disease.[1,14]

## Risks for developing MS after an initial demyelinating event

One of the most challenging aspects of pediatric MS is determining whether a child with an IDE will develop subsequent events that are consistent with MS or whether the IDE will remain a self-limiting disorder. Table 1 summarizes IDE features that are suggestive of an ultimate diagnosis of MS.[15–17] One prospective study of 296 pediatric IDE patients found that after a mean observation period of 2.9 years, 57% experienced two or more episodes of demyelination.[15] Several studies indicate that factors associated with an increased risk of a second attack include age over ten years, family history of optic neuritis (ON) or MS, intact mental status, lack of isolated myelitis, a monofocal rather than polyfocal onset, absence of seizures, and lack of meningismus. The presence of oligoclonal bands or an elevated IgG index increases the risk of MS.[15–18]

**Table 1:** Features of an initial demyelinating event of childhood which suggest subsequent attacks consistent with MS

History of optic neuritis
Family history of MS
Oligoclonal bands
Elevated IgG index
Absence of preceding infection
Absence of encephalopathy
Absence of meningismus or fever
MRI suggestive of MS

While a history of ON also increases the risk of MS, particularly when the brain MRI is abnormal;[19] the second attack confirming MS may occur many years after the initial event.[16,20]

As shown in Table 2, certain MRI findings are closely associated with MS. An increased risk of MS is associated with the presence of three out of four Barkhof criteria; namely, one or more gadolinium-enhancing lesions, three or more periventricular lesions, one or more juxtacortical lesions, and one or more infratentorial or spinal cord lesions.[21] The KIDMUS study also identified MRI criteria that were associated with an increased risk of a second demyelinating event. These criteria were the presence of one or more lesions that were perpendicular to the long axis of the corpus callosum, and the sole presence of well-defined lesions.[22] Callen criteria for MS were established for a different purpose.[23] The findings of five or more T2 lesions, two or more periventricular lesions, and one or more brainstem lesions differentiated pediatric MS from other nondemyelinating neurological disorders.

**Table 2:** MRI Criteria Associated with MS

Barkhof (21)	KIDMUS (22)	Callen (23)
At least 3 of the following:	All of the following:	At least 2 of the following:
≥ 9 T2 lesions or ≥ 1 gadolinium enhancing	≥ 1 lesion perpendicular to long	≥ 5 T2 lesions
≥ 3 periventricular	axis of corpus callosum	≥ 2 periventricular
≥ 1 brainstem	Sole presence of well-defined lesions	≥ 1 juxtacortical
≥ 1 infratentorial or spinal cord lesion		

## Clinical features of MS

Children present with a wide variety of symptoms including sensory deficits, ON, brainstem-related deficits, motor deficits, and gait disorders. Some studies suggest that children are more commonly polysymptomatic (50–70%) than adults[7,15,24] although a monosymptomatic (30–50%) presentation is not uncommon. Of the children with monosymptomatic presentation, 30% will have motor symptoms, 30% sensory symptoms, 25% brainstem symptoms, 10–22% present with ON, and 5–15% with ataxia.[7,24–26] Isolated transverse myelitis is seen in < 10%[1,4,15] and ADEM as an initial presentation of pediatric onset MS is seen in 18–29% of the patients.[11,15,16,18,27,28] Seizures are estimated to occur in 5%[26] and visual-evoked potentials were found retrospectively to be abnormal in 56% of 85 patients in whom only 40% had visual complaints in the past. Thus, ON may be underreported in pediatric MS, especially in younger children who may have difficulties in verbalizing this symptom or who have not yet started to read.[29]

Several clinical features are more common in young MS patients (those under 11 years of age).[5] These include a history of preceding infection, more frequent severe cognitive problems, seizures, optic nerve dysfunction, and brainstem or cerebellar involvement. In contrast, it is less common for younger to have isolated spinal cord presentations. Younger patients also have more confluent disease on brain MRI with lesions that tend to vanish more quickly.[11,17,30–32] One study found that those under the age of 12 years had a longer relapse-free interval and lower number of relapses in the first two years of disease compared to those older than 12 years.[6]

Fatigue that limits recreational and scholastic activities is a common symptom of pediatric MS.[26,33] Children also appear to be exceptionally vulnerable to cognitive disability. The most common impairments are complex attention, visuomotor integration, confrontation naming, receptive language, and executive function while verbal fluency tends to be relatively intact.[33] The frequency of cognitive impairment ranges in different studies from 30 to 66%.[11,33–35] Unfortunately, preliminary data suggest that as many as 70% of children decline in cognitive ability over two years.[36] This is in contrast to adults where cognitive decline is typically more gradual.

### Prognosis

Over time, children with MS develop repeated relapses and accumulate increasing disability. Annual relapse rates vary in different studies from 0.5 to 2.8,[2,8,37,38] depending on differences in prospective *vs*. retrospective design of the studies and duration of follow-up. Overall, when compared to adults, children with MS have a higher rate of relapse within the first two years of disease but progress more gradually.[7,25,32] In several retrospective studies, the time to reach an EDSS of 6.0 from diagnosis varied from 19 to 29 years.[6,14] At higher levels of EDSS, the likelihood is that the course will have transitioned to secondary progressive MS. The risk of secondary progressive MS in children (as in adults) is associated with a higher frequency of relapses and shorter intervals between attacks in the first few years of the disease.[2,7]

## Differential Diagnosis

An essential feature of the diagnosis of MS is that there is dissemination in time and space. Further, non-neurological involvement or systemic disease is absent. A more complete review of the differential diagnostic disorders and the laboratory work-up are reviewed elsewhere.[39,40]

### ADEM

The most common and perhaps the most difficult differential on first presentation is acute disseminated encephalomyelitis (ADEM). Considered a post- or parainfectious demyelinating process, this disorder also presents with multiple lesions and multiple neurological deficits.[41] A large part of the confusion stems from the lack of inclusion of a standard definition for ADEM in many publications. The International Pediatric MS Consensus definitions provide provisional diagnostic criteria for MS, ADEM, and other acquired demyelinating disorders of the central nervous system (CNS).[42] Criteria for ADEM are that the syndrome is multifocal and requires encephalopathy. However, this definition is likely to be too restrictive as some multifocal, nonencephalopathic patients will have a self-limiting disease course as would occur in ADEM.[16] Additional variants of ADEM which appear to be rare, are recurrent ADEM or multiphasic ADEM. A second inflammatory event develops in these variants. However, unlike an MS attack, encephalopathy is present and the MRI shows characteristic features of ADEM. Further, the disease is subsequently self-limiting without further events after the second episode.[30,42]

In a retrospective chart review study comparing MS and ADEM, patients with ADEM were found to be more likely to have nonspecific symptoms such as fever, headache, vomiting, meningismus, encephalopathy, and bulbar symptoms such as dysarthria and dysphagia.[43] A history of recent viral illness or vaccination is usually elicited in ADEM. Other important features include seizures, cranial neuropathies, and ON.[16,17,41] CSF studies tend to show a mild to moderate leukocytosis, elevated protein, and a lower frequency of oligoclonal bands compared to MS. Typically, the MRI in ADEM shows large (≥ 2 cm) lesions which can involve deep grey matter as well as white matter. MRI features favoring ADEM as opposed to MS, include the presence of diffuse bilateral lesions, a lack of two or more periventricular lesions, and a lack of black holes.[44] Often, a subsequent change in MRI or the clinical course reveals the correct diagnosis.[45]

### NMO

Another inflammatory disorder of the CNS that can mimic MS is neuromyelitis optica (NMO), formerly referred to as Devic's disease. The criteria for its diagnosis require optic neuritis and transverse myelitis, and either a longitudinally extensive lesion on spinal cord MRI or a positive NMO IgG antibody titer.[42] The presence of brain involvement is not uncommon in children and does not exclude the diagnosis.[46]

### Infection and other disorders

Encephalitic or meningo-encephalitic infectious etiologies must be ruled out in patients with acute presentations, particularly when there is fever and CSF leukocytosis. CNS Lyme disease may manifest with multifocal white matter lesions and a relapsing/remitting clinical course. Similar patterns may be seen with HIV encephalomyelitis, HTLV-1, neurosyphilis, progressive multifocal leukoencephalopathy (PML), Whipple's disease, subacute sclerosing panencephalitis (SSPE), and various parasites.

### Vascular and autoimmune disorders

CNS vasculitis can be difficult to distinguish from MS. The presence of beading on CT angiograms can suggest the diagnosis, as can an elevated C-reactive protein level or erythrocyte sedimentation rate (ESR). A brain biopsy may be necessary in cases of isolated CNS angiitis. Other disorders to include in the differential diagnosis are systemic lupus erythematosus (SLE), Behçet disease, neurosarcoidosis, and Sjogren's disease. Vascular diseases mimicking MS are fortunately rare in younger individuals and would include moyamoya disease and cerebral autosomal dominant arteriopathy with subcortical infarcts and leukoencephalopathy (CADASIL). In some cases of CADASIL, there is marked overlap on brain MRI with MS. The family history, stroke-like events, and migraines help lead to the correct diagnosis with confirmation of a Notch3 mutation. Other disorders to consider are Langerhans cell histiocytosis and hemophagocytic lymphohistiocytosis.

### Neoplasms

Cranial neoplasms, particularly CNS lymphoma, may mimic a tumefactive presentation of MS on neuroimaging and complicate the diagnosis.[40,47] Including MRS studies with the routine MRI might help in the differential diagnosis in these instances.

### Leukodystrophies

Leukodystrophies can be subdivided into myelination failure, delay or breakdown, and those associated with malformations. Key features are bilateral and symmetric involvement on MRI that appears fairly homogenous. Adrenoleukodystrophy and adrenomyeloneuropathy can be confusing as they show preferential involvement of the peritrigonal white matter, splenium of the corpus callosum, posterior limb of the internal capsule, the crus cerebri, and the cerebellar white matter.[40] The inclusion of very long chain fatty acids in the laboratory work-up of suspected cases leads to the correct diagnosis.

Macrocrania with white matter dystrophy suggests either Alexander disease or Canavan disease. Whereas the infantile onset of leukodystrophies is usually clearly differentiated from MS, those with a juvenile onset can overlap. For example, juvenile cases of Alexander disease may lack macrocephaly and the posterior fossa may be preferentially involved. The bilateral frontal white matter involvement may be less conspicuous. Persistent contrast enhancement can also occur. A very extensive review of the MRI appearance of white matter disorders that may be confused with MS is available elsewhere.[48] A case of Pelizaeus Merzbacher has been described which overlapped considerably with MS, both in clinical and radiological manifestations. Marked nystagmus led to testing of the proteolipid 1 protein abnormality and the correct diagnosis was established.[49] In general, progressive cognitive decline (as was present in the Pelizaeus Merzbacher case) is typical for leukodystrophies but extremely rare in MS. Degenerative and metabolic diseases that may mimic MS include metachromatic leukodystrophy, Krabbe disease, Refsum disease, vanishing white matter disease, Wilson's disease, Fabry disease, vitamin B12 deficiency, folate deficiency, vitamin E deficiency, and celiac disease.

### Mitochondrial disorders

The course for mitochondrial disorders is typically progressive and may be marked by bilateral basal ganglia involvement on MRI. Further red flags, such as visual loss, bilateral hearing deficit, short stature, ophthalmoplegia (Kearns-Sayre), cardiac involvement (Kearns-Sayre/CPEO), stroke-like events (MELAS), myoclonic epilepsy (MERRF), or bone marrow failure (Pearson), can help reveal the specific cytopathy. Brainstem involvement is a feature seen in Leigh syndrome.

## Diagnostic testing

A standard diagnostic evaluation beyond imaging and CSF should include: CBC, ESR, and ANA, whereas extended testing for specific conditions might include Lyme antibody titers, MR angiography, MR spectroscopy, evoked potentials, CSF lactate, serum vitamin levels (B12, D, E, folate), anti-Ro, anti-La, serum angiotensin-converting enzyme levels, HIV, rapid plasma reagent, HTLV-1 PCR, serum EBV and mycoplasma titers, NMO antibody, very long chain fatty acids, and GFAP mutation. Further laboratory testing for specific disorders is more thoroughly reviewed elsewhere.[39,40]

## Treatment

### Communicating the Diagnosis

Being told of the diagnosis of MS can be traumatic for the patient and family. That the prognosis is uncertain and the condition is rare contributes to the difficulty the family faces in adjustment. A number of considerations should go into the process of conveying the diagnosis. It is important to emphasize to the family that they are not alone and that online social network groups, support groups, and literature specific to the topic of pediatric MS are available.

### Management of relapses

The usual treatment for an acute relapse is corticosteroids with doses of parenteral methylprednisolone ranging from 10 to 30 mg/kg. In most instances, the maximum dose is 1000 mg administered intravenously (IV) once daily in the morning for 3–5 days; an oral prednisone taper is optional. An alternative to IV steroids is high-dose, oral prednisone (same dose as IV therapy) which in adults may be as effective as IV treatment[50] in managing acute relapses.[51] High-dose oral prednisone (up to 1200 mg/day) in adults has been found to have good bioavailability compared to IV therapy[52] but is untested in children or adolescents. Adverse effects of steroids such as insomnia as well as mood disturbances including psychosis, hyperglycemia, and hypertension need to be monitored. Prolonged steroid use can also possibly retard growth in youngsters. Despite its long-term risks, short courses of steroid therapy are reasonably safe and well tolerated by most children.

Plasmapheresis or intravenous immunoglobulin (IVIG) are options for when IV steroids fail to improve a severe relapse. The use of either modality is based on its success in a limited number of cases of children or adults.[53,54] Usually five exchanges every other day are administered. IVIG treatment can also be used at a dose of 0.4 g/kg for five days and be continued one day per month at 0.4 g/kg. This therapeutic approach has been studied in only small samples of ADEM and its variants.[55,56]

### Treatment with disease modifying therapy (DMT)

Multiple studies have demonstrated treatment of pediatric MS to be well tolerated [Table 3]. To the extent that efficacy can be established in the absence of placebo controlled trials, the body of evidence supports treatment. The bulk of studies demonstrating treatment efficacy is drawn from comparisons of pretreatment relapse frequency to that during treatment. However, a comparison of untreated to treated patients was also found to favor treatment. In this study, 197 pediatric MS patients were followed after their MS-defining event for an average of 5.5 years. A total of 24 patients began interferon therapy 3.6 months (mean period) after their relapse whereas 73 remained untreated. Those treated had a relative reduction in relapses over the subsequent first two years on therapy with a hazard ratio of 0.40, *P* < 0.01. However, the benefit was less apparent over the four years of follow-up.[57]

**Table 3:** First-Line Disease-Modifying Therapies in Pediatric MS (selected studies)

Drug	Number of children per study (N)	Duration of Treatment (months)	Most Frequent Adverse Events	Percent Decrease in relapse rate from pretreatment	Reference
IFN beta-1a 30 μg IM once a week	13	12	Flu-likesymptoms, injection-site reactions, transient abnormal liver enzymes	NA	(60)
	52	43	Flu-like symptoms, headache, myalgia	79%	(58)
	9	17	Flu-like symptoms, injection site reactions.	NA	(59)
IFN beta-1b					
0.25 mg SC every other day	43	29	Flu-like symptoms, LFT increase	50%	(62)
			Injection site reactions		
IFN beta-1a	16	41	*Flu-like symptoms,	73%	(37)
SC 22 μg or 44 μg three times per			Laboratory abnormalities		
week	51	22	Injection site reactions, flu-like symptoms, abnormal blood counts	58%	(38)
	24	44	Flu-like symptoms, injection site reactions, abnormal liver enzymes	Significant	(61)
Glatiramer Acetate	7	24	Systemic reaction	100%	(64)
20 mg SC daily	9	33	None reported	91%	(37)

* Side effects includes two patients on INF beta-1b

### Interferon (IFN)

IFN-beta 1a intramuscular (IM) injection at a dose of 30 micrograms (μg) once weekly is usually well tolerated in children with MS. In the largest study to date, a total of 53 children under the age of 16 years were started on therapy and followed for a mean of 43 ± 20 months.[58] Of this group, 19 discontinued treatment and the reasons for discontinuing were most often due to a switch to what was considered a more effective therapy (*n* = 13). Adverse events and laboratory changes were similar to those of treated adults. There was a drop in relapse rate from pretreatment to during therapy of 1.9 to 0.4 for the entire group.[58–60] The standard adult dose of 30 micrograms (μg) IM once per week has been used effectively even in some ten year-old children but half the dose is often used for very young children aged three years and younger.

Interferon beta 1a subcutaneous (SC) injection has also been successfully administered to pediatric MS patients. Treatment was well tolerated among 51 children under the age of 16 years who were started on IFN-beta 1a SC therapy.[38] The mean annual relapse rate decreased from 1.9 prior to initiation of therapy to 0.8. Adverse events were similar to those of treated adults.[58,60,61] The dose for IFN-beta 1a SC for children can be the same as adults: 44 μg three times a week with a minimum of 48 hours between each dose at a lower dose of 22 μg three times a week.[38]

A multicenter collaborative effort was completed among 43 individuals who all started on IFN-beta 1b SC therapy prior to their 18^th^ birthday.[62] There were no serious adverse events. Flu-like symptoms (35%), elevated LFTs (25%), and injection site reactions (21%) were the most frequent side effects.[62,63] Dosing was the same as in adults. The relapse frequency on therapy was lower relative to pretherapy with a mean reduction in the annualized relapse rate of 50%.[62]

### Glatiramer acetate (GA)

In a study of seven patients aged 9–16 years who were treated with GA, all did well at standard doses of 20 mg daily and without escalation.[64] In controlled clinical trials of adults, the most commonly observed adverse experiences associated with the use of GA were: injection site reactions, vasodilatation, chest pain, asthenia, infection, pain, nausea, arthralgia, anxiety, and hypertonia. These side effects have also been reported in children.

### Breakthrough disease

Unfortunately, many children treated with first-line DMTs experience breakthrough disease and need to be switched to either another first-line treatment or to second-line therapies. In a preliminary study of 164 treated children, 25% had breakthrough disease with initial DMT. Of these, eight children were switched to chemotherapy, two went on natalizumab, and two were treated with pulse IVIG.[65] Other children do well with cyclophosphamide.[66]

### Natalizumab in pediatric MS

A total of four children treated with natalizumab have been reported[67,68] who were 12–13 years of age at the time of therapy and had failed first-line DMTs (interferons and glatiramer acetate). A dose of 3–5 mg/kg of body weight was administered, leading to a decrease in disease activity and, in three patients in whom it was recorded, improved quality of life. All patients tolerated the treatment well, but none at the time of the report had been followed for more than 24 months. The potential development of PML remains a substantial concern in choosing this treatment option.

## Treatment Adherence

Compliance requires acknowledgement of the diagnosis by the family and patient, participation, and agreement in the treatment plan. Poor adherence can result from poor family dynamics, limited education of the disease, and incomplete understanding of the purpose of therapy. Moreover, a transition in responsibility occurs in adolescence, although greater compliance is seen with parental involvement.[6]

## Symptomatic therapy

There are no clinical trials of symptomatic therapy in children with MS. There have been studies showing efficacy of oral and intrathecal baclofen for spasticity in children with other neurological disorders.[69,70]

Pediatric MS is a chronic disorder with variable risk following an initial clinical attack.[66] Clearly some patients whose presentation resembles ADEM will subsequently be reclassified as MS. The differential diagnosis and clinical features differ slightly from adults with the disease and are most distinctive in the youngest patients. The management of the disease includes educating and reassuring the family, using medications to modify the disease course, and addressing the daily symptoms and psychosocial consequences of the disease.
